# Analysis of the Influence of Construction Insulation Systems on Public Safety in China

**DOI:** 10.3390/ijerph13090861

**Published:** 2016-08-30

**Authors:** Guowei Zhang, Guoqing Zhu, Guoxiang Zhao

**Affiliations:** 1 Faculty of Safety Engineering, China University of Mining and Technology, Xuzhou 221008, China; zgw119xz@126.com; 2 Key Laboratory of Building Fire Protection Engineering and Technology of Ministry of Public Security of the People’s Republic of China, Tianjin 300000, China; 3 Department of Flow, Heat and Combustion Mechanics, Ghent University, Brussels B-1000, Belium; zgq119xz@cumt.edu.cn

**Keywords:** fire spread, XPS panels, public safety, risk assessment

## Abstract

With the Government of China’s proposed Energy Efficiency Regulations (GB40411-2007), the implementation of external insulation systems will be mandatory in China. The frequent external insulation system fires cause huge numbers of casualties and extensive property damage and have rapidly become a new hot issue in construction evacuation safety in China. This study attempts to reconstruct an actual fire scene and propose a quantitative risk assessment method for upward insulation system fires using thermal analysis tests and large eddy simulations (using the Fire Dynamics Simulator (FDS) software). Firstly, the pyrolysis and combustion characteristics of Extruded polystyrene board (XPS panel), such as ignition temperature, combustion heat, limiting oxygen index, thermogravimetric analysis and thermal radiation analysis were studied experimentally. Based on these experimental data, large eddy simulation was then applied to reconstruct insulation system fires. The results show that upward insulation system fires could be accurately reconstructed by using thermal analysis test and large eddy simulation. The spread of insulation material system fires in the vertical direction is faster than that in the horizontal direction. Moreover, we also find that there is a possibility of flashover in enclosures caused by insulation system fires as the smoke temperature exceeds 600 °C. The simulation methods and experimental results obtained in this paper could provide valuable references for fire evacuation, hazard assessment and fire resistant construction design studies.

## 1. Introduction 

With the Government of China’s proposed Energy Efficiency Regulations (Code for Acceptance of Energy Efficient Building Construction GB40411-2007), external insulation systems will be mandatorily implemented in China. At present, the common external insulation materials in China mainly consist of organic insulation materials (such as extruded polystyrene (XPS panel) insulation board, expandable polystyrene (EPS panel) board and polyurethane (PU), etc.) and inorganic insulation materials (such as rock wool, inorganic fibre, etc.). Because of its low cost and good insulation performance, XPS panel is the most common insulation material in China. Although XPS panel has good insulation performance, its fire resistance is poor, which explains the nearly one hundred XPS insulation system fires that have occurred in China since 2005 [1]. When XPS panels burn, the flames may spread upward along the building’s exterior and spread into enclosures through vents (such as windows and doors). As a result, in tall buildings insulation fires are not simply limited to a single-layer or multi-layer fire, but rather will spread vertically and horizontally and eventually cause the three-dimensional combustion of the whole building. Among these external insulation fires, the China Central Television (CCTV) center fire which occurred in 2009 caused the death of a fireman and 26.5 million U.S. dollars in direct economic losses [1]. A Shanghai teacher’s apartment fire in 2010 caused 58 deaths and 25.8 million U.S. dollars in direct economic losses [1] and the Shenyang Wanxin hotel fire occurred in 2011 caused 483.8 million U.S. dollars in direct economic losses [1]. 

Frequent external insulation material fires thus cause huge casualties, large property damage and social panic and have rapidly becomes a new hot issue which has aroused great concern from a majority of fire scholars in China. Research on external thermal insulation material fires is still mainly focused on the materials’ pyrolysis and combustion characteristics using lots of cone calorimeter or medium size experiments [2,3,4,5,6,7]. Lefebvre dealt with the fire properties of flexible polyurethane foams and provided basic correlations between some existing fire test methods and the data recorded under cone calorimeter conditions [8]. Lautenberger proposed a generalized pyrolysis model which is applied to simulate the oxidative pyrolysis of white pine slabs irradiated under nonflaming conditions. Conservation equations for gaseous and solid mass, energy, species, and gaseous momentum (Darcy’s law approximation) inside the decomposing solid are solved to calculate profiles of temperature, mass fractions, and pressure inside the decomposing wood. The prosed calculations reproduce well the experimental data [9,10]. Chaos presented an approach which involves complex-step differentiation to compute the normalized first-order local sensitivity coefficients of relevant model outputs with respect to the inputs, i.e., the material properties. The proposed approach is both systematic and robust and provides sensitivity coefficients that are dynamic; that is, sensitivity values are given as a function of time for the entire pyrolysis process [11]. He also presented lots of analyses aimed at evaluating the plausibility of obtaining material properties numerically from pyrolysis data collected in a Fire Propagation Apparatus (FPA) [12]. Stoliarov presented a new computational tool, ThermaKin2D, that expands the ThermaKin model to two dimensions and combines it with a flexible analytical representation of a surface flame. This tool enables highly accurate simulations of flame spread dynamics which were verified by a series of verification exercises [13]. Lautenberger proposed a new approach to modeling soot formation and oxidation in non-premixed hydrocarbon flames which has been developed and subjected to an initial calibration. The model considers only the phenomena essential for obtaining sufficiently accurate predictions of soot concentrations to make Computational Fluid Dynamics (CFD) calculations of fire radiation feasible in an engineering context. The soot model have been embedded within a modified version of National Institute of Standards and Technology (NIST) Fire Dynamics Simulator and used for a comparison of predicted and measured temperatures, soot volume fractions, and velocities in laminar ethylene, propylene, and propane flames [14].

However, as stated above, due to the limitations of experimental funding and conditions, full-scale experiments to study the flame spread and smoke hazards of external insulation system fires are impossible to conduct. Especially the whole building in a three-dimensional fire caused by external insulation material fires is still rarely researched. With the rapid improvement of computer performance, considerable attention has been paid to fire simulation. Since the National Institute of Standards and Technology (NIST) released Fire Dynamics Simulator (FDS) in 2000, it has been a powerful tool for simulating the consequences of fire scenarios involving realistic geometries [15]. For example, Chi used thermal analysis experiments and the Fire Dynamics Simulator (FDS) to reconstruct an arson fire scene. His study was based on an arson attack which occurred in Taiwan. His study used PU foam as the experimental material and utilized thermal analysis equipment to obtain heat reaction data as input parameters for the FDS program. The results were compared and verified with the on-site fire spread and smoke debris to obtain heating temperatures which were close to the actual conditions, as well as corresponding material parameters for the reconstruction of the arson attack [16].

In this paper, the combustion characteristics of XPS panel are experimental studied and we attempt to reconstruct an actual fire scenario and provide a risk assessment of an external insulation material fire using FDS-SMV version 6 (NIST, Washington, DC, USA). The accuracy of large eddy simulation on the upward fire spread was validated by a medium-sized extruded polystyrene insulation board (XPS panel) fire experiment. Taking a nine-story building as an example, the flame spread along an external building facade is investigated by large eddy simulation. These methods and experimental data will provide a reference and support for fire simulation and fire prevention of external insulation systems.

## 2. Combustion Characteristics of XPS Panel

### 2.1. Ignition Temperature, Heat of Combustion and Limiting Oxygen Index 

ISO 871:2006 Determination of ignition temperature using a hot-air furnace, ISO 4589-2 Burning test for Oxygen Index test, and ISO1716:2002 Determination of the heat of combustion tests were separately applied to obtain the ignition temperature, heat of combustion and limiting oxygen index of XPS panel using specific instruments. By serial experiments as shown in Table 1, we find that the ignition temperature, heat of combustion and limiting oxygen index of XPS panel are separately 350 °C, 45.2 MJ/kg and 18.5%.

### 2.2. Thermo Gravimetric Analysis 

**Figure 1:**
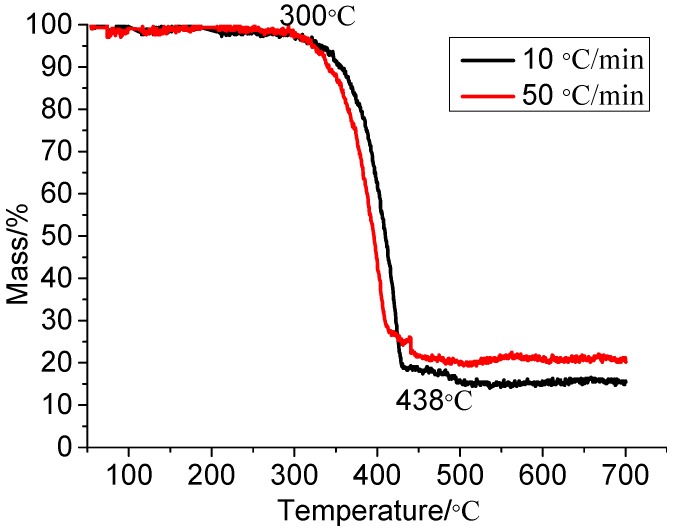
Thermogravimetry (TG) of Extruded polystyrene board (XPS panel).

A reference thermogravimetric analyzer is used to acquire the pyrolysis behavior of XPS panel. The experimental temperature range is 50 °C–700 °C, heating rates are 10 °C/min and 50 °C/min, sample mass is 1.15 mg and the reaction atmosphere is air. From the thermogravimetric analysis, as shown in Figure 1, we find that the weight loss region of XPS panel is mainly in the 300 °C–438 °C range and the mass loss in this region accounts for 75% of the total mass of XPS panel as shown in the thermogravimetry (TG) curves. These test results basically coincide with the ignition temperature (350 °C). 

### 2.3. Ignition by Thermal Radiation

**Figure 2:**
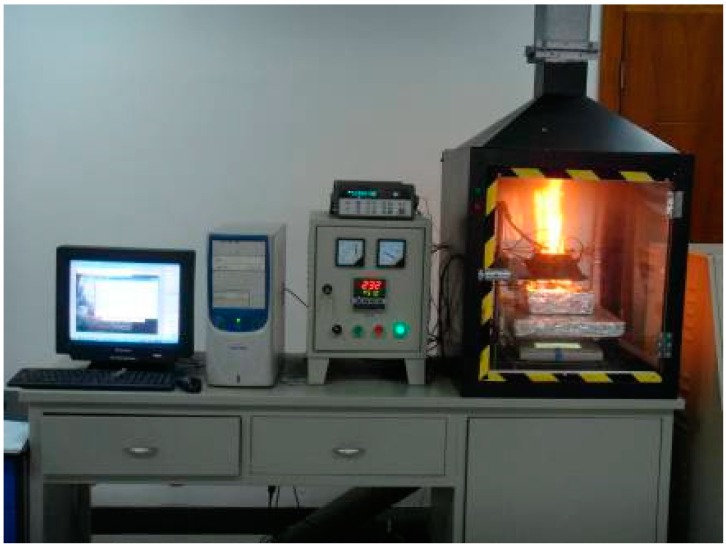
Experimental equipment of XPS panel exposure to heat radiation.

Ignition by thermal radiation experiments were conducted to study the ignition and combustion characteristics of XPS panel under different radiation heat fluxes. The experimental equipment includes a cone calorimeter, heat flux collector, temperature collector, mass loss collector and video capture, as shown in Figure 2. The samples are 10 cm (length) × 10 cm (width) × 4.5 cm (thickness), and all four sides and the bottom surface of the specimens are wrapped by inorganic fibre, thus only the top surface was exposed to the cone calorimeter which is more similar in the real fire conditions. Three groups of experiments were conducted under different radiation heat fluxes and the radiation heat flux changes linearly with time as shown in Table 2.

**Figure 3:**
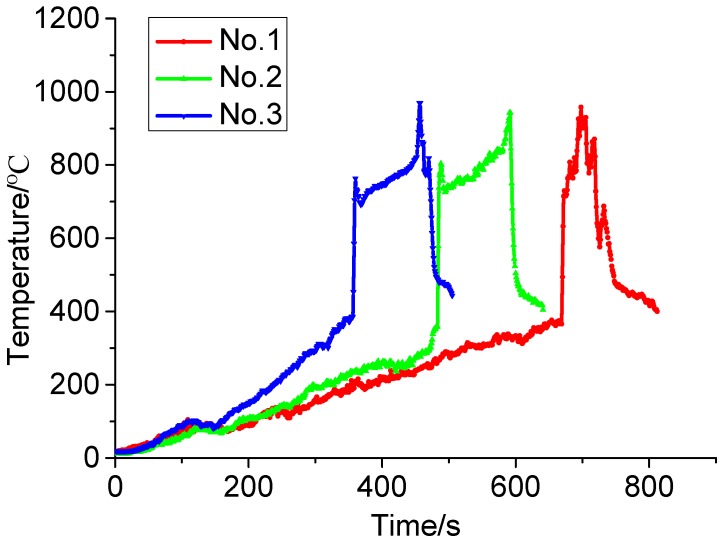
Surface temperature exposed to different heat fluxes.

K-thermocouples with high sensitivity are placed in tiny grooves at the XPS sample surface to collect the surface temperatures of XPS panels exposed to different radiation heat fluxes and the collected data is shown in Figure 3. From the collected surface temperatures and the video recorder, we can find that the surface temperature rapidly rises and reaches its maximum value once the XPS panel is ignited. With the combustion process, XPS panel eventually burn out and the surface temperature would drop correspondingly. We also find that the combustion point at which the XPS panels gets burnt is located between 336 °C and 387 °C, which is consistent with the ignition temperature obtained in Section 2.1. and referred to the reference temperature in the FDS simulation. The maximum surface temperatures in the whole combustion process are located between 933 °C and 970 °C with a mean value of 953 °C.

**Figure 4:**
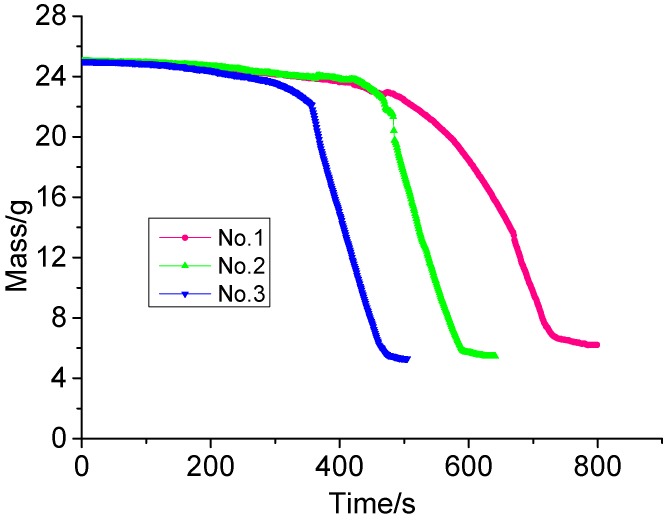
Mass of XPS panel exposed to different heat flux.

A Sartorius electronic balance collected the mass loss of XPS panels during the whole experimental process and the collection data is shown in Figure 4. We can see that mass loss rate in the experiment has three stages: slow pyrolysis stage, rapid reduction stage and relatively stable stage and that the mass of XPS panels decreases linearly in the rapid reduction stage. We also got the mass loss rate of XPS panel in the mass rapid reduction stage under different heating conditions and we find that the mass loss rate of XPS panel under different heating condition is different and that it is a constant value in the mass rapid reduction stage exposed to a linearly increasing heat flux as shown in Table 3. 

## 3. Large Eddy Simulation on Upward Fire Spread 

### 3.1. Theoretical Basis

Fire Dynamics Simulator (FDS version 6.0, NIST, Washington, DC, USA) developed by McGrattan et al. at the National Institute of Standards and Technology (NIST) was used in this research [17]. 

#### 3.1.1. Pyrolysis Models

The following assumptions of instantaneous release of gas species, local thermal equilibrium between the solid and gas components, no condensation of gas products and no porosity effects are made to undergo simultaneous reactions. Each material component may undergo several competing reactions, and each of these reactions may produce some other solid component (residue) and gaseous species according to specified yield coefficients. The local density of material component α evolves in time according to the solid phase species conservation Equation [17]:

(1) $$\frac{\partial }{\partial t}(\frac{\rho_{s,a}}{\rho_{s}(0)})=-\sum_{\beta =1}^{N_{r,\alpha }}r_{\alpha \beta }+S_{\alpha }$$

where *N_r,_*_α_ is the number of reactions for material α, *r*_αβ_ is the rate of reaction β in units of 1/s, and *ρ_s_*(0) is the initial density of the material layer. *S*_α_ is the production rate of material component α as a result of the reactions of the other components. The reaction rates are functions of solid and gas phase conditions and calculated as a combination of Arrhenius and power functions:

(2) $$r_{\alpha \beta }={\left(\frac{\rho_{s,a}}{\rho_{s}(0)}\right)}^{n_{s},\alpha \beta }A_{\alpha \beta }\exp(-\frac{E_{\alpha \beta }}{RT_{s}}){\left[X_{O_{2}}(x)\right]}^{n_{O_{2}},\alpha \beta }\max{\left[0,S_{\mathrm{thr},\alpha ,\beta }(T_{s}-T_{\mathrm{thr},\alpha \beta })\right]}^{n_{t},\alpha \beta }$$

The production term *S*_α_ is the sum over all the reactions where the solid residue is material α:

(3) $$S_{\alpha }=\sum_{\alpha^{\prime }=1}^{N_{m}}\sum_{\beta =1}^{N_{r,\alpha^{\prime }}}\nu_{\alpha ,\alpha^{\prime }\beta }r_{\alpha^{\prime }\beta }$$

where $v_{\alpha ,\alpha^{\prime },\beta }$ is the yield of component α from reaction β of component α’. The chemical source term in the heat conduction equation is:

(4) $$q_{s,c}^{\prime \prime \prime }(x)=-\rho_{s}(0)\sum_{\alpha =1}^{N_{m}}\sum_{\beta =1}^{N_{r,\alpha }}r_{\alpha \beta }(x)H_{r,\alpha \beta }$$

where *H_r_*_,αβ_ is the heat of reaction.

#### 3.1.2. Combustion Model

For most applications, FDS uses a combustion model based on the mixing-limited, infinitely fast reaction of lumped species. Lumped species are reacting scalar quantities that represent a mixture of species. For an infinitely-fast reaction, reaction species in a given grid cell are converted to product species at a rate determined by a characteristic mixing time, τ_mix_. The heat release rate per unit volume is defined by summing the lumped species mass production rates times their respective heats of formation [17]:

(5) $$q^{\prime \prime \prime }=-\sum_{\alpha }{m^{\prime \prime \prime }}_{\alpha }\Delta h_{f,\alpha }$$

where *h*_f,α_ is heat of formation of species α. 

#### 3.1.3. Radiation Model

The net contribution form thermal radiation in the energy equation is defined by [17]:

(6) $$\begin{array}{l} q_{r}^{\prime \prime \prime }\equiv -\nabla \cdot q_{r}^{\prime \prime }(x)=\kappa (x)[U(x)-4\pi I_{b}(x)]\\ U(x)=\int_{4\pi }I(x,s^{\prime })ds^{\prime } \end{array}$$

where κ(*x*) is the absorption coefficient, *I*_b_(*x*) is the source term, and *I*(*x,s*) is the solution of the radiation transport equation (RTE) for a non-scattering Gray gas.

### 3.2. Experimental Work

**Figure 5:**
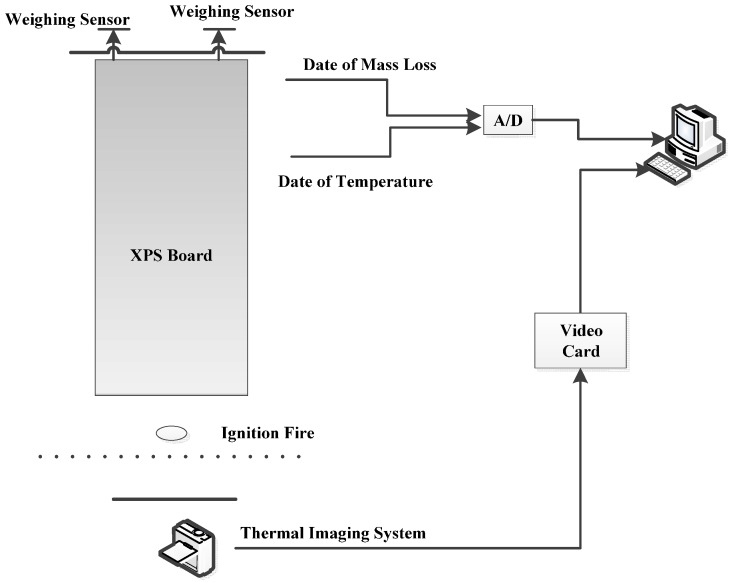
Schematic diagrams of experimental apparatus.

**Figure 6:**
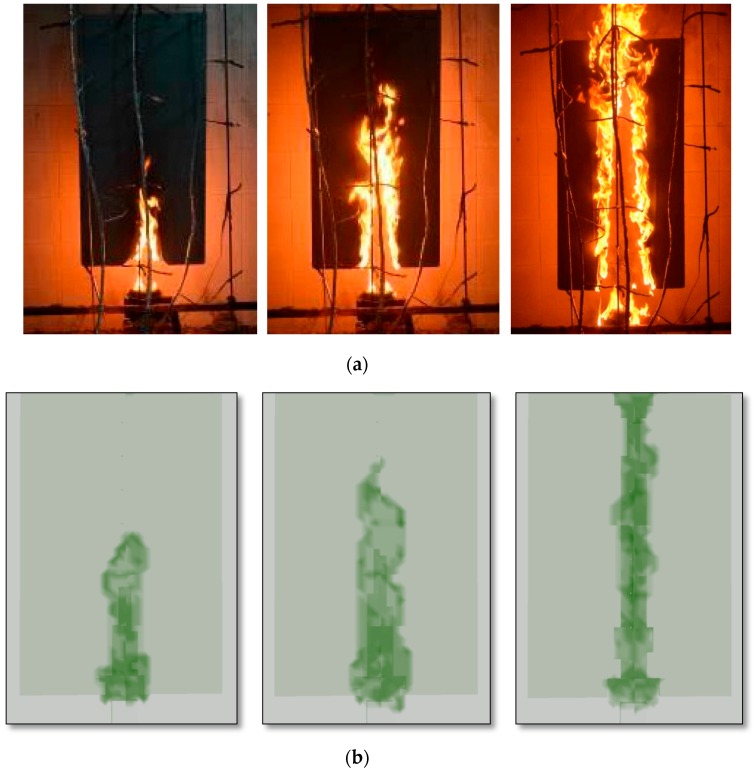
(**a**) Upward flame in experiment; (**b**) Upward flame (>200 kW/m^3^) in FDS simulation.

A medium-sized XPS fire experiment is conducted to validate the accuracy of our large eddy simulation on upward fire spread. The experimental sample, 110 cm (length) × 60 cm (width) × 4.5 cm (thickness), is hung in air as shown in Figure 5. Two high sensitivity K-thermocouples are placed in tiny grooves at the XPS panel surface to collect the surface temperatures and a weight sensor is used to record the mass loss rate of XPS panels during the whole experiment process. It is found that the flame spread rapidly with a ribbon burn pattern once the XPS panel is ignited and the vertical spread speed is faster than the horizontal spread speed as shown in Figure 6a.

### 3.3. FDS Input Data

#### 3.3.1. Geometry

The domain in FDS is constructed as close to the XPS panel experiment as stated above. The 4.5 cm thick, 60 cm wide, and 110 cm high XPS slab is hung in air. Meanwhile, the ignition source in the experiment is a diesel oil pool fire. In the FDS model a 4.5 cm long × 4.5 cm wide “burner” is created as an ignition source in the bottom of domain with same size. By referring the heat release rate of diesel oil pool fire, the heat release rate of burner surface is 850 kW/m^2^ [18]. 

#### 3.3.2. Grid Resolution

As we all know, grid size is a key factor in FDS simulation to obtain the features combustion. FDS shows sensitivity to grid size in many applications [19,20,21,22,23]. A smaller grid size is preferred for a better simulation, but if the grid is too small, performing the simulation will consume too much computer memory. Therefore, we referred to literature and used the following formula to set the grid size [24]:

(7) $$D^{*}={\left[\frac{Q}{\rho_{\infty }C_{\infty }T_{\infty }\sqrt{g}}\right]}^{2/5}$$

where *Q*, $\rho_{\infty }$, $C_{\infty }$ and $T_{\infty }$ are, respectively, the total heat release rate (kW), the density at ambient temperature (kg/m^3^), the specific heat of gas at ambient temperature (kJ/kg. K) and the ambient temperature (K). In above experiment, the mean mass loss rate in a time period is 1.5 g/s. Referring to LIU Wanfu’s experiments, the combustion efficiency of XPS panel is 0.67 [25]. Thus the maximum heat release rate in the fire experiment is 45.4 kW. As it takes account of both fine grids and the computation time, a 2.0 cm grid size is chosen. The chosen grid size is finer than the suggested grid size proposed in VTT working papers [26].

#### 3.3.3. Material Properties

Sensitivity to material properties in FDS predictions can be seen in the FDS-related works [27,28]; thus, it is crucial to use reliable values for material properties. Input parameters, such as the thickness, density, specific heat, thermal conductivity, heat of combustion, the heat of reaction, reference temperature, pyrolysis range and heating rate are obtained from the above tests and reference experiments. 

### 3.4. Results and Discussion

**Figure 7:**
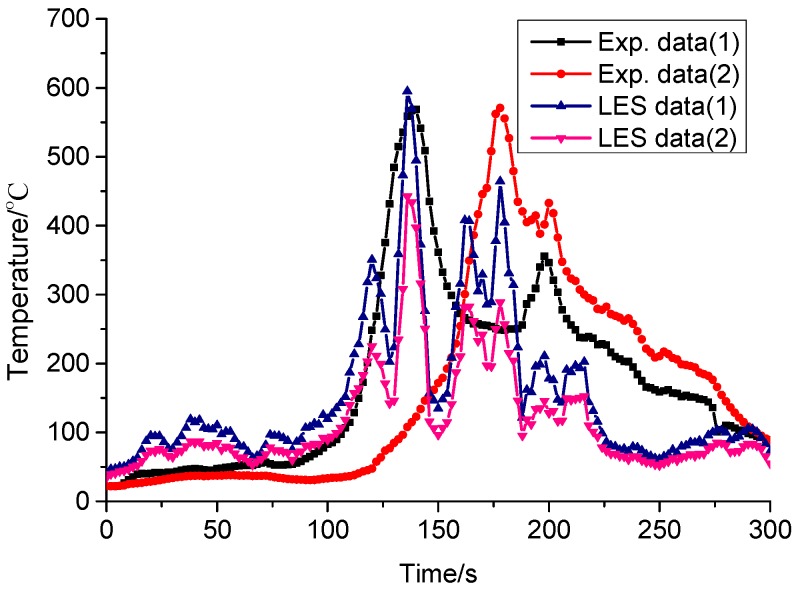
Surface temperatures in the experiment and LES simulation.

The upward flame spread along the XPS panel is presented in Figure 6a, from which we can see that the flame spreads along the XPS panel following a ribbon burn pattern. Due to the fact the flame convection directly heats the XPS panel during the fire spread process, the flame spread in the vertical direction is faster than that in the horizontal direction. The flame spread simulated by FDS version 6.0 is presented in Figure 6b. Comparing these two figures, we can see that the simulated burning patterns and flame spread are similar to those in the fire experiment. Figure 7 presents the surface temperatures in the experiment and Large eddy simulation (LES simulation). From this figure, we can see that the simulated surface temperature basically coincides with the experimental data and that the simulated temperature is higher and more sensitive than the experimental data as a result of using thermocouples which may be not sensitive enough. Meanwhile, the weight sensor recorded the mass loss rate of XPS panels during the whole experiment process. By analyzing this data, we find that the mean mass lose rate is 1.5 g/s during the period between the XPS ignition and flame spreading all over surface. Thus the mean heat release rate in this period is:

(8) Q=φ×m×ΔH

where φ is combustion efficiency, LIU Wanfu has given a suggested value 0.67 for XPS panel’s combustion efficiency [25]. Thus the mean heat release rate is Q = 0.67 × 1.5 × 45.20 kW which well agrees with the simulated data values of 40.2 kW. When the flame spreads all over surface, the XPS panel is burning violently and dripping appears frequently. At this time, the mass lose rate recorded by the balance has no reference value. 

As discussed above, we can see that the fire spread speed, surface temperature and mass loss rate obtained by large eddy simulation methodology basically coincide with the experimental data. Thus one conclusion could be made is that the large eddy simulation could well predict the XPS upward fire spread.

## 4. Fire Hazard Assessment Based on LES

As discussed above, large eddy simulation methodology could predict well the upward fire spread. Thus in this section the large eddy simulation methodology is applied to a representative nine-story building to make a fire hazard assessment of external insulation materials fires. 

### 4.1. Fire Model

**Figure 8:**
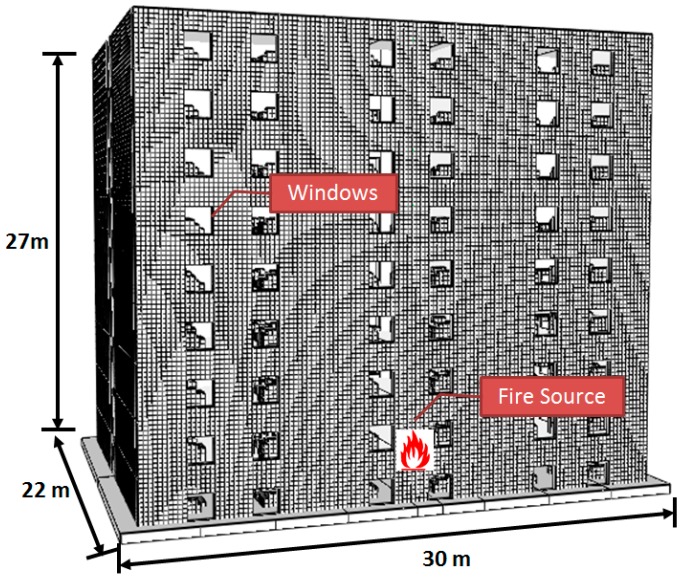
Numerical configuration of a representative nine-story apartment.

Due to the average building size in China, the researched building is a representative nine-story apartment with 30 m (length) × 22 m (width) × 27 m (height), as shown in Figure 8. 

There are six enclosures in each floor and some furniture combustibles (sofas, chairs, beds), two windows (1.0 m × 2.0 m) and one door (1.5 m × 1.8 m) in each enclosure. All the windows and doors included in the FDS geometry are open. A fire source located at XPS external insulation materials of a central enclosure 3.0 m away from ground. Supposing welding slag ignitew advertising cloth during the welding operation, we can assume that the ignition fire source, a *t*^2^ fire, reaches its maximum heat release rate of 250 kW at 180 s and then moves out after 180 s. XPS’ material properties and input parameters are the same as discussed above, as shown in Table 4, and other main components’ parameters in the model are set in Table 5. A mixing-controlled combustion model is applied in the simulation. Applying the mixing-controlled combustion model easily causes numerical diffusion, thus a fine grid size is preferred. Referring to the grid resolution above and the grid size proposed in VTT working papers shown in Table 6 [26], in the nine-story building fire simulation, the grid size applied 0.20 m × 0.20 m × 0.20 m. 

### 4.2. Fire Spread Upward along the Building's Exterior

**Figure 9:**
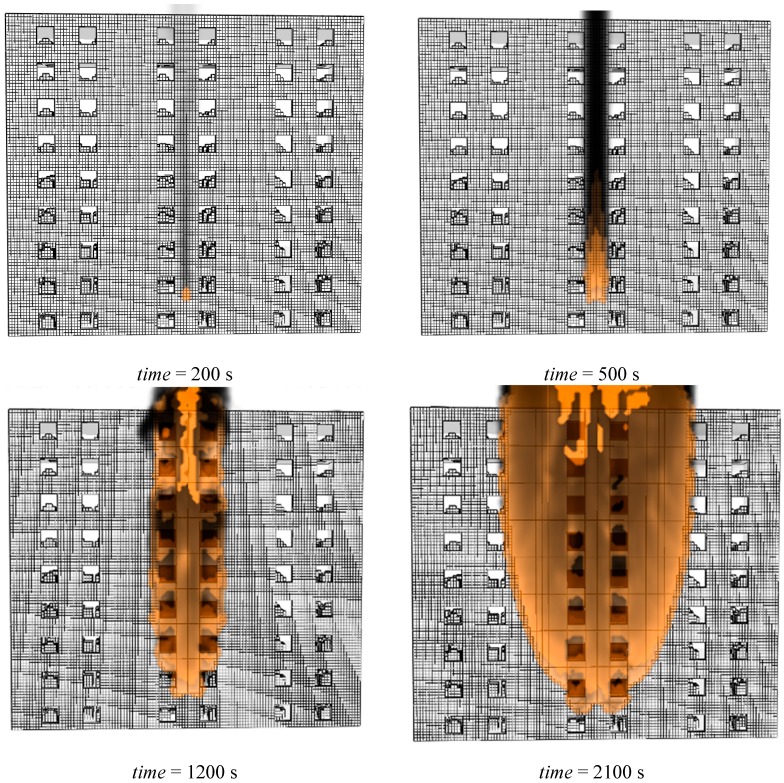
Fire spread upward along the building's exterior predicted by LES.

**Figure 10:**
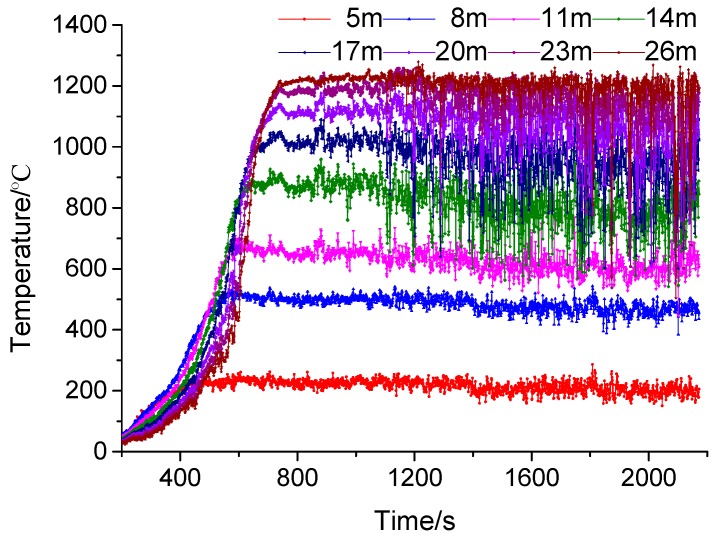
Surface temperatures of building’s exterior.

**Figure 11:**
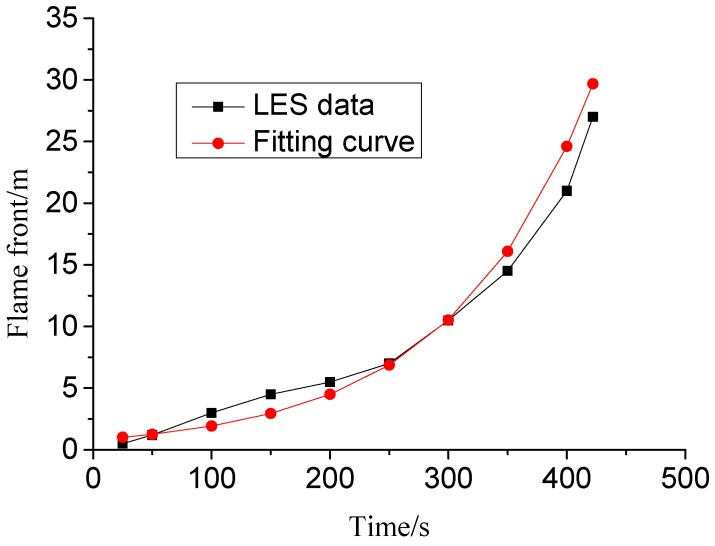
Flame spread in the vertical direction.

From the simulation results, we find that the XPS external insulation materials are ignited by the ignition fire source (stated in Section 4.1), then fire spreads upward with an accelerated speed along the building’s exterior and spreads into enclosures through windows and a three-dimensional combustion fire of the whole building ocurrs, which is identical to a fire accident occurred in China as shown in Figure 9. We also find that the external insulation material fire spread in the vertical direction is faster than that in the horizontal direction and it only takes 422 s for the flame front to reache the building roof and that the smoke temperature of the building’s exterior during the fire distributes non-homogeneously (the more distance away from the fire source, the higher the smoke temperature is, as shown in Figure 10) which indicates that the fire becomes more violent duringn the fire process. The flame spread in the vertical direction is shown in Figure 11. By fitting, we find that the relationship between the flame spread speed and time can be expressed as Equation (9),

(9) $$s(t)=8.214\times 10^{-1}e^{0.0085t}$$

where *s*(*t*) is the flame front position (m) and *t* is time after ignition (s).

### 4.3. Temperature Profile

The *SFPE Handbook of Fire Protection Engineering* has given criteria for smoke temperatures which cause deaths during fires. The critical criteria of smoke temperature is 60 °C at 2 m height from the ground [28]. In this section, this critical criteria is applied to make quantitative assessment of danger time caused by smoke temperatures.

**Figure 12:**
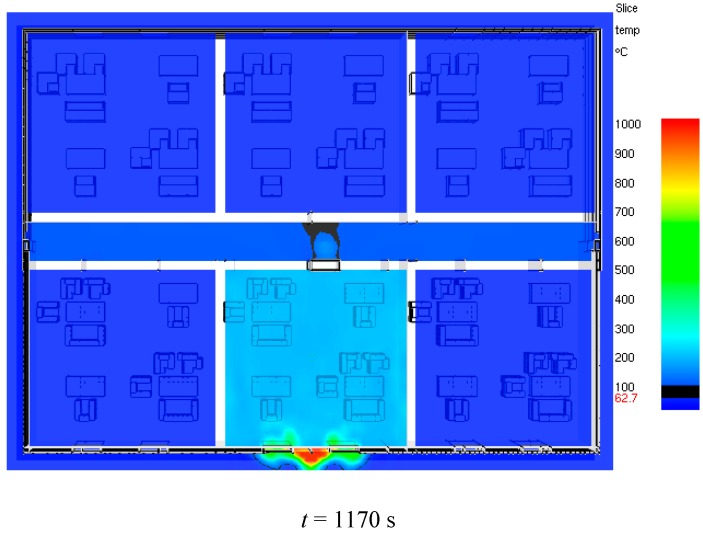

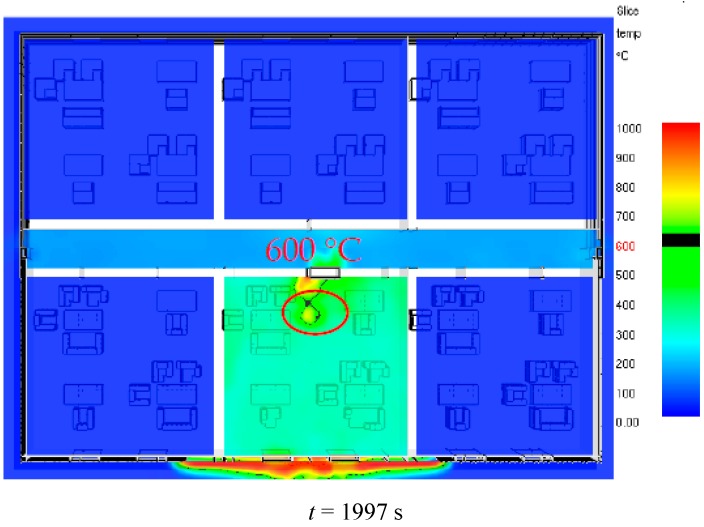
Smoke temperature distribution at eighth floor obtained by LES.

Danger times caused by smoke temperature in corridors are evaluated as shown in Table 7 and we can see that the most dangerous floor is the fourth floor (6 m above the fire source) and the danger time in this floor is only 999 s. We can also see that the more distance away from the fourth floor (6 m above the fire source), the longer the danger times is. Meanwhile, smoke temperatures in enclosure 2# on the eighth floor exceed 600 °C at 1997 s as shown in Figure 12 and we can believe that there is flashover risk in this enclosure which is dangerous for firefighters [28,29]. A flashover is the near-simultaneous ignition of most of the directly exposed combustible material in an enclosed area. When certain organic materials are heated, they undergo thermal decomposition and release flammable gases. Flashover occurs when the majority of the exposed surfaces in a space are heated to their auto ignition temperature and emit flammable gases. Thus firefighters should note the flashover risk in external insulation materials fire rescue operations.

## 5. Conclusions

Combustion characteristics and fire risk of exterior insulation materials fire are researched in this paper. Ignition temperature, heat of combustion, limiting oxygen index, TG curve and ignition by thermal radiation are separately studied experimentally firstly. Based on these experimental data, large eddy simulation is applied to reconstruct an actual fire scenario and provide a risk assessment of fire spread along an external building facade made of polystyrene insulation board. Simulation methods and research results obtained in this paper could provide valuable references for public evacuation, hazard assessment and fire protection design. Based on this research, the following conclusions can be drawn: 

Ignition temperature, heat of combustion and limiting oxygen index of XPS panel are separately 350 °C, 45.2 MJ/kg and 18.5%. Weight loss region of XPS panel is mainly in the 300 °C–438 °C region and the mass loss in this region accounts for 75% of the total mass. XPS mass loss rate under thermal radiation has three stages: slow pyrolysis stage, rapid reduction stage and relatively stable stage and it decreases linearly in the rapid reduction stage. Moreover, the mass loss rate of XPS panel under different heating condition is different and it is a constant value in the mass rapid reduction stage exposed to a linearly increasing heat flux. 

A medium-sized XPS fire experiment was conducted to validate the accuracy of the large eddy simulation on upward fire spread. We find that the fire spread speed, surface temperatures and mass loss rate obtained by large eddy simulation methodology coincide with the experimental data, thus we believe large eddy simulation could predict well the XPS upward fire spread. Then the large eddy simulation methodology is applied to a representative nine-story building to provide a risk assessment of fire spread along an external building facade made of polystyrene insulation board. We find that the simulated fire spread area is identical to that of fire accidents occurred in China and that the external insulation material fire spread in the vertical direction is faster than that in the horizontal direction. Moreover, we also find that there is a possibility of flashover indoors as the smoke temperature exceeds 600 °C in enclosures. Thus we can believe that the insulation systems used in construction could seriously influence public safety in case of a fire. It only takes 422 s for the flame front to reach a 27 m high building roof and the fire becomes more violent and exposes evacuees and firefighters to more hot smoke and flashover during the fire process. Firefighters should note the risk of flashover during external insulation material fire rescues. 

## Figures and Tables

**Table 1:** Material properties of XPS panel.

Material Properties	1# Sample	2# Sample	3# Sample	Mean Value
Ignition temperature/°C	348	349	353	350
Heat of combustion/(MJ/kg)	45.5	45.1	45.0	45.2
Oxygen index/%	18.7	18.5	18.3	18.5

**Table 2:** Radiation heat flux of each experiment.

Identification (ID) of Experiments	Radiation Heat Flow
NO.1	q=0.0381t (*t* is time (s) and q is heat flux (kW))
NO.2	q=0.0604t (*t* is time (s) and q is heat flux (kW))
NO.3	q=0.0753t (*t* is time (s) and q is heat flux (kW))

**Table 3:** Fitting of mass loss rate curve in the mass rapid reduction stage under different heating condition.

Heating Condition (kW)	Fitting Curve for Mass Loss Rate of XPS Panel	Mass Loss Rate (g/s)
q=0.0381t	m=−0.1122t+11.80	0.1122
q=0.0604t	m=−0.1330t+19.61	0.1330
q=0.0753t	m=−0.1477t+22.10	0.1477

**Table 4:** Material properties and input parameters.

Input Parameters	Value	Input Parameters	Value
Thickness (m)	0.45	Density (kg/m^3^)	25
Specific heat (kJ/kg·k)	1.34	Ignition temperature	350
Combustion heat (kJ/kg)	45,200	Reference temperature	369
Conductivity (w/m/k)	0.10	Pyrolysis range (°C)	138
Radiative fraction	0.35	Heat of reaction (kJ/kg)	1750

**Table 5:** Main parameters of components in the model.

Main Components	Density kg/m^3^	Heat Capacity kJ/(kg·°C）	Heat of Combustion MJ/kg	Conductivity W/(m·°C)	Ignition °C
Foam	40	1.00	30.0	0.05	350
Concrete	2280	1.04	--	1.8	--

**Table 6:** Suggested grid size for representative fire experiments proposed in VTT working papers [26].

Representative Fire Test	Heat Release Rate (kW)	Suggested Grid Size (cm)
Cone calorimeter	2	2.0
Single burning item (SBI) test	50	7.5
Room corner test	500	20

**Table 7:** The dangerous time caused by smoke temperature in different floors.

Floor	Second Floor	Third Floor	Fourth Floor	Fifth Floor	Sixth Floor	Seventh Floor	Eight Floor	Tenth Floor
Available evacuation times	1786 s	1014 s	999 s	1026 s	1045 s	1157 s	1170 s	1214 s
